# Comparative Toxicity of *Helicoverpa armigera* and *Helicoverpa zea* (Lepidoptera: Noctuidae) to Selected Insecticides

**DOI:** 10.3390/insects11070431

**Published:** 2020-07-10

**Authors:** Fernando R. da Silva, Dario Trujillo, Oderlei Bernardi, Jose Carlos Verle Rodrigues, Woodward D. Bailey, Todd M. Gilligan, Daniel Carrillo

**Affiliations:** 1 Tropical Research and Education Center, University of Florida, 18905 SW 280th, St. Homestead, FL 33031, USA; silvafr@yahoo.com.br; 2 Center for Excellence in Quarantine & Invasive Species, University of Puerto Rico (UPR), San Juan, PR 00926-1118, USA; darioxtru@gmail.com (D.T.); jose_carlos@mac.com (J.C.V.R.); 3 Department of Plant Protection, Federal University of Santa Maria, CCR-Building 42-Room 3233, Campus-Camobi, Santa Maria 9710590, RS, Brazil; oderleibernardi@yahoo.com.br; 4 United States Department of Agriculture, Animal and Plant Health Inspection Service, Plant Protection and Quarantine, Science and Technology, Miami, FL 33158, USA; woodward.d.bailey@usda.gov; 5 United States Department of Agriculture, Animal and Plant Health Inspection Service, Plant Protection and Quarantine, Science and Technology, Fort Collins, CO 80526, USA; todd.m.gilligan@usda.gov

**Keywords:** corn earworm, insecticide, invasive species, Old World bollworm, resistance

## Abstract

Until recently, the Old World bollworm (OWB) *Helicoverpa armigera* (Hübner) and the corn earworm *Helicoverpa zea* (Boddie) (Lepidoptera: Noctuidae) were geographically isolated. Both species are major pests of agricultural commodities that are known to develop insecticide resistance, and they now coexist in areas where *H. armigera* invaded the Americas. This is the first study to compare the susceptibility of the two species to conventional insecticides. The susceptibility of third instar *H. armigera* and *H. zea* larvae to indoxacarb, methomyl, spinetoram, and spinosad was determined using a diet-overlay bioassay in a quarantine laboratory in Puerto Rico. Mortality was assessed at 48 h after exposure for up to eight concentrations per insecticide. Spinetoram exhibited the highest acute toxicity against *H. armigera*, with a median lethal concentration (LC_50_) of 0.11 µg a.i./cm^2^, followed by indoxacarb and spinosad (0.17 µg a.i./cm^2^ for both) and methomyl (0.32 µg a.i./cm^2^). Spinetoram was also the most toxic to *H. zea* (LC_50_ of 0.08 µg a.i./cm^2^), followed by spinosad (0.17 µg a.i./cm^2^) and methomyl (0.18 µg a.i./cm^2^). Indoxacarb was the least toxic to *H*. *zea*, with an LC_50_ of 0.21 µg a.i./cm^2^. These findings could serve as a comparative reference for monitoring the susceptibility of *H. armigera* and *H. zea* to indoxacarb, methomyl, spinetoram, and spinosad in Puerto Rico, and may facilitate the detection of field-selected resistance for these two species and their potential hybrids in areas recently invaded by *H. armigera*.

## 1. Introduction

The noctuid moths Old World bollworm (OWB), *Helicoverpa armigera* (Hübner, 1809) and corn earworm, *Helicoverpa zea* (Boddie, 1850), are major lepidopteran pests attacking crops worldwide. The latter is restricted to the New World and attacks more than 120 host species in 29 plant families [1,2,3,4]. *Helicoverpa armigera* feeds on more than 180 hosts in 70 plant families, and it is widely distributed in Europe, Africa, Asia, and Oceania [5,6,7,8,9]. It was first reported in the New World in 2013, infesting soybean and cotton fields in Brazil [10]; a year later, it was detected in Argentina and Puerto Rico [11,12]. The two species have similar external morphologies, and their identification requires the labor-intensive dissection of male genitalia and/or molecular analysis [13,14,15]. Surveys in Puerto Rico revealed low *H. armigera* population densities in areas where *H. zea* is found, suggesting that it is still in an early stage of invasion. A phylogenetic analysis using the cytochrome b (Cytb) region determined that *H*. *armigera* from Puerto Rico has two haplotypes. One of these is the second most frequently found worldwide, the other is only present in the north western region of Brazil, suggesting that the population in Puerto Rico may have originated in South America [16].

The larvae of both *Helicoverpa* species commonly feed on the reproductive tissue of their host plants [17]. The last instar of *H. armigera* can account for more than 85% of the total damage caused by the larval stage on cotton [18,19]. In the U.S.A., *H. zea* attacks more than 30 crops, and it is considered one of the most injurious pests of tomato, corn, and cotton, contributing to recurring losses of around one billion USD per year [20,21,22,23,24]. Even higher annual losses are attributed to *H. armigera,* estimated at 5 billion USD worldwide [19,25,26]. Approximately 50% of the total insecticides applied in India and China are used to control *H. armigera* [27]; in Brazil, its damage was estimated at 2 billion USD during the 2012/2013 season [28].

*H. armigera* and *H. zea* utilize similar resources and ecological niches [29,30]. Recent studies have suggested that *H. zea* derived from *H. armigera* and lost genes related to detoxification [31,32], as well as certain genes that confer resistance to insecticides [33,34,35,36,37]. However, the two species can mate with each other and produce fertile progeny that may have resistance levels that are unlike those of the parental species [36,38,39]. Wild hybrids of *H*. *armigera* and *H*. *zea* have been reported in Brazil [39], and a few individuals were detected in Puerto Rico in 2014/2015 that are presumed to be hybrids based on molecular analysis (Gilligan, T.M.; unpublished).

These species have exhibited reduced susceptibility to groups of insecticides, including carbamates, organophosphates, pyrethroids, and *Bacillus thuringiensis* proteins. The unsatisfactory control of *H. armigera* with the pyrethroids deltamethrin, cypermethrin and fenvalerate was reported in Brazil [37,40]. Previous studies have also reported a high resistance frequency to pyrethroids in Australian populations [41]. Reports of pyrethroid resistance in *H. zea* started in the early 1990’s [42,43]. Consequently, insect resistance management (IRM) programs have been adopted around the world to delay or prevent resistance development in these two species.

Potential changes in the susceptibility of *H. armigera* and *H. zea* to conventional insecticides represent a major threat to agriculture in areas with established populations of these species and their potential hybrids. Hence, it is important to develop susceptibility tests and monitor changes in resistant ratios on target populations for these two species. This study compared the susceptibility of a population of *H. armigera* from Brazil, the presumed epicenter of the infestation in South America, and a population of *H. zea* from Puerto Rico to four commercial insecticides. Our study aimed to support proactive integrated pest management programs in Puerto Rico and other areas recently invaded by *H. armigera*.

## 2. Materials and Methods

### 2.1. Insect Populations

The *H. armigera* colony was established with five larvae and 30 pupae from a laboratory population maintained at the University of São Paulo, Piracicaba, SP, Brazil. This population was originally collected from soybean in Mato Grosso, Brazil, and maintained in the laboratory for five generations before being shipped to the quarantine facility at the Center for Excellence in Quarantine and Invasive Species (CEQIS) in Puerto Rico on the 4 February 2017 (Puerto Rico Department of Agriculture permit number OV-1617–03 and United States Department of Agriculture, Animal and Plant Health Inspection Service permit number P526P-15-04600). No information is available on the previous exposure of this population to insecticides.

The *H. zea* colony was started with larvae collected in Isabela, Puerto Rico, on unsprayed pigeon pea on the 11 November 2015 (18°0′34′′ S; 66°53′33′′ W), and it was replenished multiple times with additional specimens collected from corn in the same area to maintain colony vigor. There are no large row crop operations in this area of Puerto Rico, and the *H. zea* individuals were collected from unsprayed experimental plots at the University of Puerto Rico—Isabela Agricultural Experimental Station.

### 2.2. Species Confirmation

Morphological and molecular tools (real-time PCR analysis) were used to determine species. Male genitalia were extracted and analyzed following the methods described by Brambila [44]. Males and females of both *Helicoverpa* species were identified by real-time PCR with specific primers for the internal transcribed spacer 1 (ITS1) region, as well as the sequencing of cytochrome c oxidase subunit I (COI) and Cytb regions [14]. Both populations were maintained at the CEQIS and were shared with other laboratories to be used as a reference for future studies and screening of other populations.

### 2.3. Rearing Procedure

Larvae were reared individually in 30 mL transparent plastic cups containing an artificial moth diet (Frontier Agricultural Sciences, Product # F9630B, Newark, DE, USA) until pupation. Pupae were transferred to Petri dishes with autoclaved vermiculite (Vigoro^®^, Lake Forest, IL, USA). One day before adult emergence, pupae were placed in white 5-gallon (19 L) plastic buckets (15.6” × 11.8”) with lids lined with cheesecloth (DeRoyal, BIDF2012380-BX, Powell, TN, USA) that served as an oviposition substrate. Adult moths were provided with a 10% sucrose solution. The oviposition substrate was replaced daily and stored in 3.8 L Ziploc^®^ (Racine, WI, USA) bags with thin strips of diet. Third instar larvae were transferred to cups with an artificial diet (described above). Colonies were maintained at 25 ± 2 °C, 65 ± 9% relative humidity (RH), and a 14:10 light:dark (L:D) photoperiod, with the exception of female pupae. They were placed in incubators (Sanyo^®^, MLR-351H, New York, NY, USA) set at a lower temperature (22 ± 1.5 °C, 75 ± 4% RH, and a 14:10 L:D photoperiod) to synchronize their emergence with that of the adult males [45,46]. Prior to this study, *H. armigera* and *H. zea* were reared for 11 and 24 generations, respectively.

### 2.4. Insecticides

The variability of response of *H. armigera* and *H. zea* to spinetoram and spinosad (allosteric modulators of nicotinic acetylcholine receptors, IRAC MoA (Insecticide Resistance Action Committee Mode of Action) group 5), indoxacarb (voltage-dependent sodium channel blockers, IRAC MoA group 22A), and methomyl (acetylcholinesterase inhibitor, IRAC MoA group 1A) were evaluated (Table 1).

### 2.5. Bioassays

The same artificial diet used to maintain the colonies was used in the bioassays. Bioassay cups placed on 30-well trays were filled with 1 mL of diet per well (4.3 cm top diameter, 3.3 cm bottom diameter, and 3 cm height). A 100 ppm A.I. stock solution of each insecticide was serially diluted to obtain the test concentrations. Triton X-100 (0.1%, Sigma Aldrich, MO, USA) was used as a surfactant to obtain a uniform distribution over the diet surface. The control treatment was composed of distilled water and a surfactant. Up to eight concentrations of each insecticide were tested for each species. The insecticides were applied to the diet surface with a replicating pipette, ultimately delivering 140 µL per cup (equivalent to 20 µL per cm^2^). The diet surface area in each cup was 7.0 cm^2^. After a 30 min drying period, one *H. armigera* or *H. zea* third instar larva was transferred to each cup using a fine paintbrush (AIT Art^®^, 10/0, Danbury, CT, USA). The cups were closed with a perforated lid that allowed for gas exchange and stored in a climate chamber (25 ± 2 °C, 65 ± 9% RH, and a 14:10 L:D photoperiod). The bioassays were repeated four times for each species, and each replication consisted of 30 larvae per concentration. Larvae were inspected after 48 h and recorded as dead if there was no movement when gently touched with a fine paintbrush.

### 2.6. Statistical Analysis

Mortality data were subjected to Probit analysis (PROC PROBIT, SAS Institute 2000) [47] to estimate the lethal concentrations (LC_50_ and LC_90_—insecticide concentrations (µg a.i./cm^2^) required to kill 50% and 90% of larvae, respectively, in 48 h) and their confidence intervals (CIs). A likelihood test was conducted to determine whether the response of the two species differed significantly in either slope or intercept [48]. Pairwise comparisons were performed, and significance was declared when CIs did not overlap [48,49]. Significant differences among slopes were determined through a likelihood ratio test for parallelism and equality [48]. For each insecticide, the tolerance ratio (TR) was determined by dividing the LC_50_ and LC_90_ of the more susceptible species by the corresponding parameter of the other species.

## 3. Results

The Indoxacarb-induced mortality of third instar larvae for both *H. armigera* and *H. zea* was concentration-dependent (Table 2). Concentrations ranging from 0.0051 to 1.60 µg a.i./cm^2^ caused 4–100% mortality. The LC_50_ of indoxacarb on *H. armigera* was 0.17 µg a.i./cm^2^, and the LC_90_ was 1.70 µg a.i./cm^2^; they were slightly higher for *H. zea* at 0.21 and 2.64 µg a.i./cm^2^, respectively. The tolerance ratios for the LC_50_ and LC_90_ values were similar at 1.24 and 1.55-fold, with *H. zea* exhibiting a slightly lower susceptibility. The response for both species were also statistically similar, as indicated by the 95% fiducial limits overlap.

Methomyl produced the greatest variation in response between the species (Table 2). Concentrations from 0.0051^−3^ to 2.88 µg a.i./cm^2^ caused mortality ranging from 5% to 100% in both species; however, the LC_50_ and LC_90_ for *H. armigera* were 0.32 and 3.20 µg a.i./cm^2^, respectively, which were much higher than those for *H. zea* (0.18 and 1.88 µg a.i./cm^2^, respectively). The tolerance ratios were lower than 1.8-fold, indicating a similar response of these species to methomyl.

Spinosad and spinetoram also induced high mortality for both species (Table 2). Concentrations ranging from 0.0051 to 1.60 µg a.i./cm^2^ caused 3–100% mortality. The spinosad LC_50_ value for both species was 0.17 for µg a.i./cm^2^. The spinetoram LC_50_ values were 0.11 and 0.08 µg a.i./cm^2^ for *H. armigera* and *H. zea*, respectively. In contrast, a lower LC_90_ of spinosad was detected for *H. armigera* (1.48 µg a.i./cm^2^) than on *H. zea* (3.30 µg a.i./cm^2^). The LC_90_ of spinetoram was similar for both species (0.67 and 0.68 µg a.i./cm^2^ for *H. armigera* and *H. zea*, respectively). The tolerance ratios, based on LC_50_, were 1.0- and 1.4-fold to spinosad and spinetoram, respectively.

## 4. Discussion

This is the first study to compare the response of *H. armigera* and *H. zea* to broad spectrum and selective insecticides. Earlier studies with biological and chemical insecticides have evaluated the two species separately due to their former geographic isolation [1,2,3,4,5,6,7,8,9,10]. Among the insecticides tested in this study, high levels of resistance of *H. armigera* to methomyl were reported in Pakistan [50,51,52], India [53,54], and Greece [55]; in contrast, low levels of resistance were reported in populations from Spain and Turkey [56,57], and no resistance was reported in invasive populations of *H. armigera* in Brazil [58]. In the U.S.A., a low frequency of resistance alleles to methomyl in *H. zea* populations from Virginia was reported [59]. However, Vemula et al. [60] found variations in the tolerance of *H. zea* to methomyl between bean crop seasons in Texas and New Mexico.

Populations of *H. armigera* from Australia were highly susceptible to indoxacarb, with toxicity ratios between 1.2 and 3.5 among several populations. The most tolerant strain had an LC_50_ value of 0.518 mg/mL [61]. However, follow-up studies identified field populations with up to a 198-fold resistance [62]. In addition, a population of *H. armigera* from China subjected to 11 generations of selection to indoxacarb resistance decreased its susceptibility by 4.43-fold (LC_50_ increased from 5.93 to 26.25 mg L^−1^) [63]. *Helicoverpa assulta* Guenée, another related species, also demonstrated resistance to this pesticide in China [64]. In south-eastern U.S.A., first instar larvae of *H. zea* under high indoxacarb pressure were very susceptible, with LC_50_ values ranging from 1.05 to 1.54 ppm using diet overlay bioassays [65], and no evidence of resistance was found in cotton fields in the U.S.A. [66].

The use of spinosyns, which include spinosad and spinetoram, to control *Helicoverpa* spp. has increased in recent years. Spinetoram has been reported to have high efficacy against *Helicoverpa* species under field conditions [67,68]. Interestingly, spinosad resistance is associated with a reduced fitness, as reflected in prolonged egg, larval, and pupal periods and decreased pupal survival and overall fecundity [66]. However, a remarkable variation in *H. armigera* population susceptibility, especially to spinosad, was reported in Pakistan [69], and populations in China developed more than 20-fold resistance after 15 generations [70]. In contrast, low levels of resistance to spinosad were reported in Pakistan [71] and populations of *H. armigera* from two intensive cotton growing areas in India [72]. The results in our study are similar to Pereira’s [73], who found two-fold variations in the susceptibility to spinosad among different populations of *H. armigera* in Brazil, thus suggesting low levels of resistance; unfortunately, after a few years of exposure, resistance increased, resulting in a 22% survival (LC_99_). *Helicoverpa zea* susceptibility to spinosad is also variable by population. In the U.S.A., high LC_50_ values were obtained for *H. zea* third instar larvae [65]; the authors suggested that this was due to the reduced rates used in cotton systems. In contrast, López Jr. et al. [74] indicated that this pesticide is highly effective against *H. zea* adults in insecticide-baited traps in the southern U.S.A.

Our results indicated that spinetoram is highly toxic to both *Helicoverpa* species. This insecticide is considered an important alternative for controlling *Helicoverpa* pests, especially for Cry1Ac-resistant populations [68]. Xie et al. [67] found spinetoram to be effective against *H. armigera*, inducing high mortality rates and sublethal effects similar to spinosad in *H. armigera* populations from China [66]. Visnupriya and Muthukrishnan [75] also reported low LC_50_ values for spinetoram on *H. armigera*, ranging from 1.94 to 5.20 ppm. There have been few reports of *Helicoverpa* species resistance to spinetoram; nevertheless, if usage patterns and exposure to sublethal concentrations of spinetoram increase, selection for resistance to it is also likely to rise [68].

The diet overlay bioassay is a valuable tool for monitoring changes in susceptibility to insecticides in *Helicoverpa* species [66,76]. This bioassay has been used to evaluate a range of insecticides (permethrin, thiodicarb, chlorfenapyr, cypermethrin, di-ßubenzuron, cyanamid, emamectin, benzoate, and spinosad) on larvae of *Diatraea saccharalis* (Fabricius, 1794), *H. armigera, H. zea,* and *Spodoptera frugiperda* (Smith, 1797), among other lepidopteran pests [77,78,79,80]. The overlay diet bioassay may better simulate the field application of insecticides than commonly used techniques such as a diet-incorporation bioassay. It allows for the even distribution of insecticides over the diet surface, thus simulating field deposition of insecticides over the surface of the larval feeding substrate. There is a caveat, as Roush et al. [81] pointed out that laboratory colonies are formed from a small number of individuals that lack the high frequency of alleles that confer field populations with resistance to insecticides, so results for laboratory populations could differ from field-selected resistance.

## 5. Conclusions

The recent establishment of *H. armigera* populations in the *H. zea* native range, as well as the potential for hybridization of these two species, may form a *Helicoverpa* complex in the Western Hemisphere. Monitoring the susceptibility of this complex to insecticides is essential for implementing IRM programs to prevent control failures. We present data on an invasive population of *H. armigera* from Brazil and a population of *H. zea* from Puerto Rico that showed similar responses to indoxacarb, methomyl, spinetoram, and spinosad. These populations can be used as a reference for future studies to develop baselines for monitoring field-selected resistance in *Helicoverpa* species.

## Figures and Tables

**Table 1:** Commercial insecticides used to compare the susceptibility of *Helicoverpa armigera* and *Helicoverpa zea* in Puerto Rico.

Active Ingredient	Trade Name	Insecticide Group	Manufacturer	Concentration Range (µg a.i./cm^2^)
Indoxacarb	Avaunt^®^ 30WG	Oxadiazines	FMC, Philadelphia, PA, USA.	0.0051–1.60 µg a.i./cm^2^
Methomyl	Lannate^®^ LV	Carbamate	Corteva Agriscience, Wilmington, DE, USA.	0.0051–2.88 µg a.i./cm^2^
Spinetoram	Radiant^®^ SC	Spinosyn	Corteva Agriscience, Wilmington, DE, USA.	0.0051–1.60 µg a.i./cm^2^
Spinosad	Entrust^®^ SC	Spinosyn	Corteva Agriscience, Wilmington, DE, USA.	0.0051–0.90 µg a.i./cm^2^

**Table 2:** Concentration–mortality response (lethal concentration (LC); µg a.i./cm^2^) of the third instar *Helicoverpa* larvae exposed to the insecticides overlaid on artificial diet.

Insecticide	Species	*n*	Slope ± SE	LC_50_ (95% FL) *^a, b^*	LC_90_ (95% FL) *^a, b^*	χ^2 *c*^	Df *^d^*	TR_50_ *^e^*	TR_90_ *^e^*
Indoxacarb	*H. armigera*	960	1.27 ± 0.21 a	0.17 (0.09–0.31) a	1.70 (0.73–3.06) a	13.26	5	-	-
	*H. zea*	960	1.17 ± 0.09 a	0.21 (0.17–0.27) a	2.64 (1.74–4.56) a	2.25	5	1.24	1.55
Methomyl	*H. armigera*	960	1.28 ± 0.32 a	0.32 (0.05–0.55) a	3.20 (1.49–4.82) a	10.01	4	1.78	1.70
	*H. zea*	840	1.26 ± 0.33 a	0.18 (0.02–0.56) a	1.88 (0.60–4.99) a	11.03	4	-	-
Spinosad	*H. armigera*	840	1.37 ± 0.24 a	0.17 (0.07–0.34) a	1.48 (0.66–3.00) a	8.96	4	1.00	-
	*H. zea*	960	0.99 ± 0.16 a	0.17 (0.09–0.26) a	3.30 (1.97–7.94) a	8.01	5	-	2.23
Spinetoram	*H. armigera*	960	1.64 ± 0.30 a	0.11 (0.06–0.17) a	0.67 (0.37–2.81) a	13.46	5	1.38	-
	*H. zea*	1080	1.35 ± 0.18 a	0.08 (0.04–0.12) a	0.68 (0.40–1.62) a	9.73	6	-	1.01

*^a^* LC_50_ and LC_90_ are the insecticide concentrations (µg a.i./cm^2^) required to kill 50% and 90% of larvae in 48 h. *^b^* LC_50_ and LC_90_ values designated by different letters within a column are significantly different from each other through a nonoverlap of 95% fiducial limits. The significance of differences among slopes was determined by a likelihood ratio test of equality followed by pairwise comparisons using nonoverlapping fiducial limits. *^c^* Chi-square significant (*p* < 0.05). *^d^* Degrees of freedom. *^e^* Tolerance ratio (TR) = (LC_50_ or LC_90_ of the lower susceptible species)/(LC_50_ or LC_90_ of the other species).
